# The Essential Role of 1-Butyl-3-Methylimidazolium-Based Ionic Liquids in the Development of Transparent Silica-Filled Elastomer Systems

**DOI:** 10.3390/ma13194337

**Published:** 2020-09-29

**Authors:** Małgorzata Kuśmierek, Bolesław Szadkowski, Anna Marzec

**Affiliations:** Institute of Polymer and Dye Technology, Faculty of Chemistry, Lodz University of Technology, Stefanowskiego 12/16, 90-924 Lodz, Poland; malgorzata.kusmierek@dokt.p.lodz.pl (M.K.); boleslaw.szadkowski@dokt.p.lodz.pl (B.S.)

**Keywords:** elastomer composites, nitrile rubber, transparent composites, ionic liquids, silica

## Abstract

In this paper, we present the design of reinforced silica-filled elastomer composites exhibiting a high transparency, high mechanical performance in static and dynamic conditions, and improved electrical conductivity. Two different imidazolium ionic liquids (ILs) were used with increasing loads: 1-butyl-3-methylimidazolium bis(trifluoromethylsulfonyl)imide (BMIMTFSI) and 1-butyl-3-methylimidazolium tetrachloroaluminate (BMIMAlCl_4_). The composites were prepared in a two-roll mill. The influence of the ILs on the dispersion of the silica in the nitrile rubber (NBR) matrix was assessed by scanning electron microscopy (SEM). The presence of ILs in the NBR/SiO_2_ systems improved the crosslink density and ionic conductivity of the composites. Their mechanical properties and aging stability remained almost unchanged, at a very satisfactory level. Greater crosslinking was observed for the NBR/SiO_2_ composites containing BMIMAlCl_4_, due to its catalytic effect on the efficiency of interface crosslinking reactions. We found the optimal formulation for obtaining transparent reinforced NBR/SiO_2_ composites. The application of 2.5 phr of BMIMAlCl_4_ resulted in a high transparency in the case of NBR composites filled with 30 phr of silica.

## 1. Introduction

Elastomers constitute an extremely large class of polymer materials based on carbon chemistry. They are characterized by a high strain resistance, nonlinear stress–strain curves, strain hardening, and high shape recovery [1,2]. They are easily modified by the addition of functional additives (such as fillers), which can extend their ranges of application [3]. However, to achieve the performance required of matrix materials is a special challenge, since improving certain matrix properties may lead to the deterioration of others. For example, the tensile strength can be improved by adding a filler to an elastomer, but this can impair the optical transparency [4,5]. A variety of transparent and thermochromic materials have been reported in the literature [6,7]. Such materials have found applications in fields including biotechnology, electronic data storage, and temperature-sensitive coatings [8,9].

Recently, Das et al. [10] designed sulfur-cured styrene-butadiene (SBR) elastomers that, when compounded with specific amounts of layered double hydroxide (LDH) minerals, exhibited thermotropic properties, including thermoreversible transparency, i.e., the transparent sample becomes opaque in warm conditions and regains transparency at room temperature. In our previous work [11], we reported that the addition of high levels of LDH to an carboxylated nitrile rubber (XNBR) matrix with Mg/Al at a ratio of 2:1 as a filler and curing agent produced composites exhibiting thermoreversible transparency. The application of aluminum-rich LDH resulted in opaque materials, whereas XNBR composites filled with Mg-rich LDH were transparent, but did not show thermochromic behavior.

Nitrile rubber (NBR) is a copolymer of synthetic rubber made of acrylonitrile (ACN) and butadiene. It is particularly well-known for its excellent heat performance and high chemical resistance against oils and chemicals, as well as its good mechanical properties [12,13]. Das et al. [14] obtained transparent NBR composites using a modified curing system. An LDH filler was used to deliver zinc ions as accelerators of the vulcanization process, as well as stearate anions, which functioned as activators. Simultaneously, the mineral sheets acted as a nanofiller to reinforce the rubber matrix, totally replacing the separate zinc oxide (ZnO) and stearic acid conventionally used in rubber blends. The NBR rubber crosslinked with the sulfur and modified LDH zinc oxide in the absence of ZnO, producing a high degree of transparency that would have been impossible to obtain using the traditional ZnO, sulfur, and stearic acid curing system.

Ionic liquids (ILs) are liquids consisting entirely of ions with melting points below 100 °C. Their excellent properties, such as their very low vapor pressures and wide temperature ranges in the liquid phase, make ILs ideal candidates for use as non-volatile solvents, replacing volatile organic solvents [15,16]. Given their low vapor pressure and non-flammability, as well as their high chemical and thermal stability, ILs can have multiple functions in the preparation of polymer composites. In particular, they can help to ensure a homogeneous filler distribution and increase the ionic conductivity. However, the presence of ILs may affect other properties of rubber compounds, such as their elastic or antibacterial properties [17,18,19,20,21,22,23]. To date, there have been no studies on the impact of ILs on the transparency of NBR blends cured with a sulfur cure system.

Here, we report the successful design of an optically transparent, easily fabricated elastomer, characterized by a good elasticity and high mechanical strength due to the addition of ILs. We prepared NBR materials enhanced by a silica filler, in the presence of hydrophilic 1-butyl-3-methylimidazolium tetrachloroaluminate (BMIMAlCl_4_) and hydrophobic 1-butyl-3-methylimidazolium bis(trifluoromethylsulfonyl)imide (BMIMTFSI). The choice of these ILs was based on their high ionic conductivity and different hydrophilic/hydrophobic characteristics. The influence of the room-temperature ILs on key properties, such as the curing behavior, mechanical properties, ionic conductivity, and morphology, was studied.

## 2. Materials and Methods

### 2.1. Materials

Acrylonitrile–butadiene rubber NBR (EUROPRENE) containing 28 wt% of acrylonitrile and characterized by a Mooney viscosity (ML_1 + 4_ at 100 °C = 45) was sourced from Torimex Chemicals Ltd. (Lodz, Poland). The NBR composites were filled with fumed silica Aerosil 380 supplied by Evonik Degussa GmbH (Essen, Germany). The rubber mixtures were cured using sulfur (Siarkopol, Tarnobrzeg, Poland). Mercaptobenzithiazole (MBT), obtained from Siarkopol (Tarnobrzeg, Poland), was used as an accelerator. Zinc oxide (Huta Będzin, Poland) and stearic acid (Sigma Aldrich, GmbH, Saint Louis, MO, USA) were used as activators of the vulcanization process. The ILs, 1-butyl-3-methylimidazolium bis(trifluoromethylsulfonyl)imide and 1-butyl-3-methylimidazolium tetrachloroaluminate, with a purity of above 98%, were provided by Sigma Aldrich.

### 2.2. Methods

Acrylonitrile-butadiene rubber composites were mixed in an open laboratory two-roll mill under the following conditions: Friction ratio, 1:1.1; temperature, 40 °C; and diameter and length of the rolling mill, D = 200 mm and L = 450 mm, respectively. The rubber compositions were as follows: NBR, 100 phr; sulfur, 2 phr; mercaptobenzothiazole, 2 phr; zinc oxide, 5 phr; stearic acid, 1 phr; Aerosil 380, 30 phr; and ILs in two different load ratios, 2.5 and 5 phr. Due to the fact that the used ionic liquids are very sensitive to moisture, ILs were immediately added to the elastomer mixture. The compounds were molded at 160 °C in a hydraulic press under 15 MPa of pressure for the optimum cure time, as measured using a MonTech D-RPA 3000 Moving Die Rheometer, according to the ASTM D5289 standard. The curing characteristics of the rubber composites were determined according to the following parameters: optimum cure time, τ_90_; scorch time, t_02_; minimum elastic torque, M_min_; maximum elastic torque, M_max_; and increment of elastic torque ΔM = M_max_−M_min_, obtained from kinetic curves. After vulcanization, the crosslink density was determined by equilibrium swelling. Vulcanized samples were weighed to measure their initial mass, immersed in toluene for 48 h at room temperature, and then weighed again to measure their mass after swelling. After drying for another 48 h at 60 °C, the samples were weighed once more to measure their final mass. The crosslinking density was calculated on the basis of Flory–Rehner’s Equation (1) [24]:

(1) $$\nu_{e}=\frac{\ln\left(1-V_{r}\right)+V_{r}+\mu V_{r}^{2}}{V_{o}\left(V_{r}^{\frac{1}{3}}-\frac{V_{r}}{2}\right)},$$

where *υ_e_* is the crosslink density, *V_r_* is the volume fraction of the elastomer in swollen gel, and *V*_0_ is the molar volume of the solvent (mol/cm^3^). The *μ* elastomer–solvent interaction parameters were *μ*_0_ = 0.381 and *β* = 0.671. The morphology of the tensile fracture surfaces of the polymer composites was observed using a Zeiss Ultra Plus microscope (Zeiss/LEO, Germany). Before the scanning electron microscopy (SEM) measurements, the vulcanizates were immersed in liquid nitrogen. Following breakage, the surface of the fracture was coated with a carbon layer. Spectrophotometric measurements were performed using a Konica Minolta Sensing, Inc. CM-3600d spectrophotometer (Osaka, Japan). The color of the composites was analyzed using the CIE L*a*b* color space system, where L* is the lightness variable, coordinate a* indicates where the color falls along the red/purple-green/blue axis, and coordinate b* indicates where the color falls along the blue/purple-yellow axis. The total color change ΔE was calculated according to Equation (2):

(2) $$\Delta E\;=\;\sqrt{{\Delta L}^{2}+{\Delta a}^{2}+{\Delta b}^{2}}.$$

The Shore A hardness of the NBR composites was measured using a Zwick Roell Group durometer (Ulm, Germany), according to the PN-EN ISO 868:2005 standard. The strain–stress behavior was investigated using a Zwick Roell ZWICK 1435 testing machine at room temperature with a crosshead speed of 500 mm/min, until breakage. The tensile strength (TS) and elongation at break (Eb) were measured from five different dumbbell-shaped samples of each composite and presented as the average value. Simulation of thermooxidative ageing was performed for 3 weeks at 70 °C in a Bruckner laboratory dryer (Leonberg, Germany). Subsequently, the mechanical properties of the samples were investigated and compared with the initial values. The ageing coefficient *K* was calculated according to Equation (3):

(3) $$K=\frac{{\left(\mathrm{TS}\cdot \mathrm{EB}\right)}_{\;\mathrm{after}\;\mathrm{ageing}}}{{\left(\mathrm{TS}\cdot \mathrm{EB}\right)}_{\;\mathrm{before}\;\mathrm{ageing}}}$$

Dynamic mechanical analysis of vulcanized samples was conducted on a Q 800 Dynamic Mechanical Analyzer (TA Instruments, Greifensee, Switzerand), operating in tension mode with the following parameters: Frequency, 10 Hz; temperature range of heating from −80 to 100 °C; and heating rate, 2 °C/min. The electrical conductivity was tested using an MIC-1000 insulation resistance meter coupled with EP-1 measuring electrodes. Prior to the analysis, the surfaces of the samples were purged using hexane. Based on the results of surface and volume resistivity measurements, the conductivity of the elastomer composites was calculated using Equation (4):

(4) $$\sigma_{s}=\frac{1}{\rho_{s}}$$

where σ_s_ is the surface conductivity (S) and ρ_s_ is the surface resistivity (Ω) calculated from Equation (5):

(5) $$\rho_{s}=81.68\;R$$

## 3. Results

### 3.1. Rheometric Measurements, Crosslink Density, and Transparency Effect

**Figure 1:**
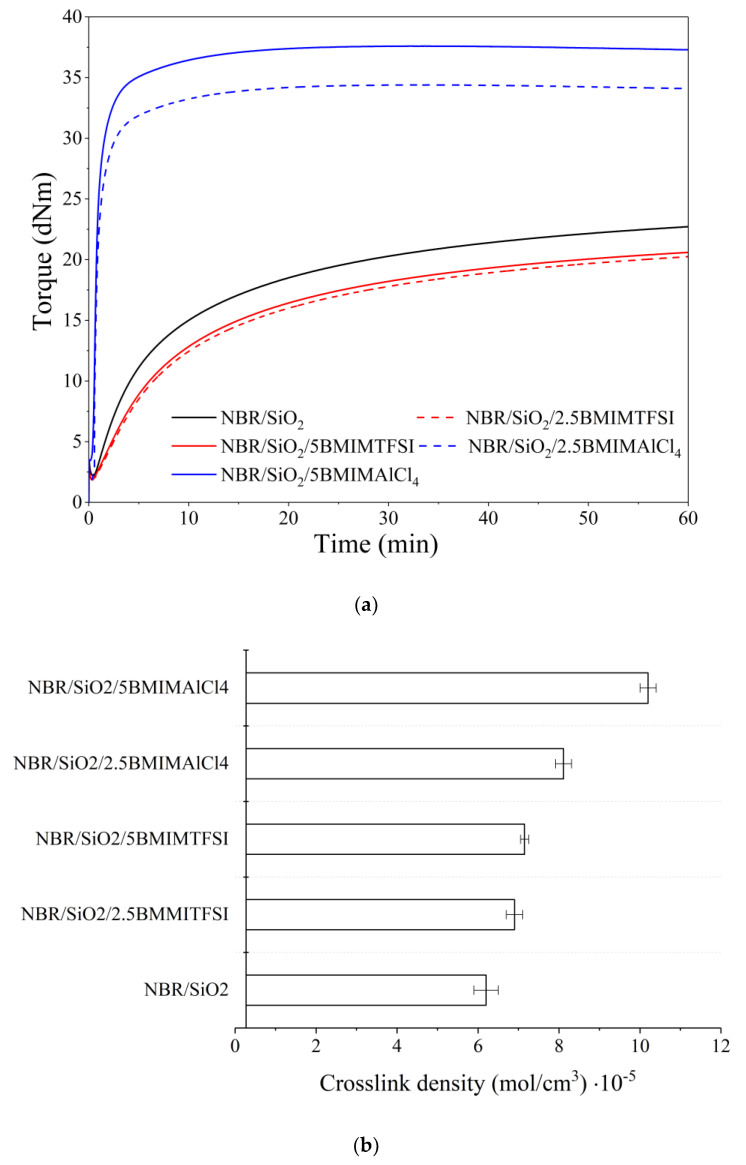
Rheometric curves for silica-filled nitrile rubber (NBR) composites with different ionic liquids (**a**) and crosslink densities of the resulting NBR vulcanizates (**b**).

The effects of the ILs on the rheological behavior and curing effectiveness of the silica-filled NBR compositions were evaluated based on rheometric and crosslink density measurements. Typical rheograms with corresponding rheometric data are presented in Figure 1a and Table 1. Crosslink density values obtained from solvent-swelling measurements are shown in Figure 1b. From Table 1a, it can be noted that the incorporation of ILs into the NBR-filled elastomer reduced the minimum toque moment. This can be attributed to the plasticizing effect of the ILs in the rubber systems [25]. According to the literature [26], the presence of ILs in a polymer matrix decreases the molecular friction and the entanglement of macromolecular chains.

Figure 1a and Table 1 also reveal that the applied ILs had a significant effect on the torque parameter (∆M), indirectly determining the increase in crosslinking of the composite. Whereas the application of BMIMTFSI did not cause significant changes compared to the reference NBR, the presence of BMIMAlCl_4_ resulted in a visible enhancement of the ∆M parameter, which increased from 20.55 dN/m for NBR up to 34.15 dN/m for NBR/5BMIMAlCl_4_. This indicates a significant improvement in the crosslink density. The incorporation of BMIMAlCl_4_ also contributed to a considerable reduction in the scorch time and optimum cure time. This can be explained by the catalytic activity of the ILs in elastomer systems cured with sulfur-based crosslinking systems [27]. On the other hand, the application of BMIMTFSI prolonged the scorch time and optimum cure time compared to the NBR/SiO_2_ system. The presence of BMIMITFSI showed lower activity in the curing process in the composites and decreased the torque values, which can be ascribed to the plasticizing effect of this IL. Our previous study has reported similar results for this type of ionic liquid in a polymer system cured with a sulfur crosslinking system [28].

Figure 1b shows the crosslink density of the silica filled-NBR composites containing ILs. In comparison to the reference NBR, the presence of imidazolium ILs generally increased the crosslink density value, especially in the case of BMIMAlCl_4_. It may be assumed that the ILs had a catalytic effect on the efficiency of interface crosslinking reactions [29]. Furthermore, it is possible that the ILs promoted the dispersion of the silica filler and other curatives in the NBR matrix, while also enhancing the filler–polymer interactions, as reflected by the higher crosslink densities of these samples. Maciejewska et al. [29] reported that the application of different ILs in nitrile rubber composites can lead to significant improvements in the crosslink density of vulcanizates, due to the more uniform dispersion of the filler and other components in the curing system. We observed such effects in our previous studies of ILs in different elastomer systems [28,30,31]. In the present study, significantly higher values for the crosslink density were observed in the case of NBR/BMIMAlCl_4_ (8.1·10^−5^ mol/cm^3^ for 2.5 phr and 10.2·10^−5^ mol/cm^3^ for 5 phr) compared to NBR/BMIMTFSI (6.9·10^−5^ mol/cm^3^ for 2.5 phr and 7.2·10^−5^ mol/cm^3^ for 5 phr). This corroborated the results of rheometric measurements.

**Figure 2:**
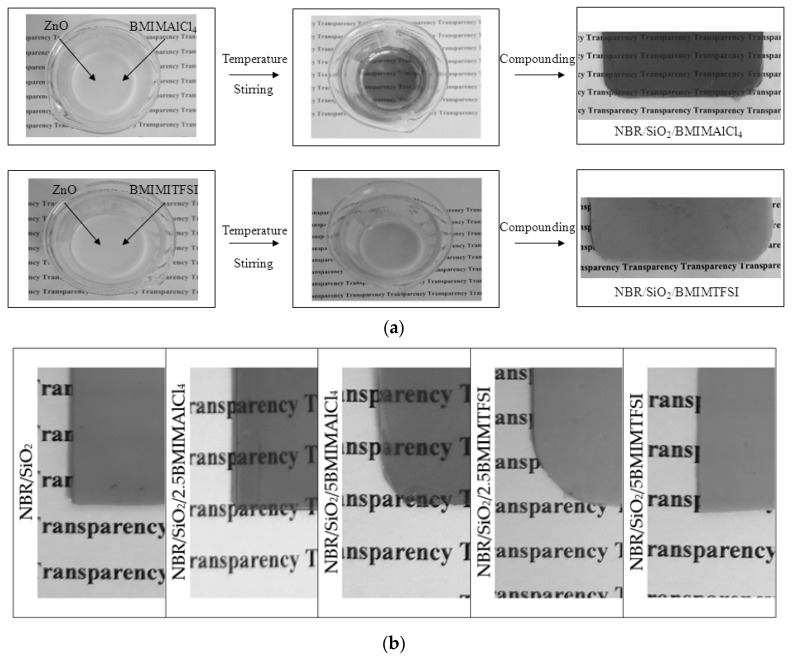
Effect of imidazolium ionic liquids: 1-butyl-3-methylimidazolium tetrachloroaluminate (BMIMAlCl_4_) and 1-butyl-3-methylimidazolium bis(trifluoromethylsulfonyl)imide (BMIMTFSI) with different anions on the transparency of NBR composites (**a**) and changes of the transparency (**b**).

Surprisingly, the composites containing BMIMAlCl_4_ became transparent after the vulcanization process (Figure 2). Because this effect was not observed for composites containing BMIMTFSI, it was concluded that the AlCl_4_ anion plays a dominant role in producing a transparent effect in this type of rubber system. This mechanism seems be related to the ability of BMIMAlCl_4_ to dissolve ZnO particles, which are applied as components in the curing formulation. It was previously reported that many oxides such as ZnO, PbO_2_, and Cu_2_O exhibited an appreciable solubility in choline-based ionic liquids [32,33]. In this work, we tested solutions of ZnO with both ILs (Figure 2), and found that heating only in the presence of BMIMAlCl_4_ produced a transparent solution.

Changes in the colorimetric parameters confirmed that increasing the concentration of BMIMAlCl_4_ caused a higher transparency, as can be seen from Table 2. The total color difference ΔE for composites containing 2.5 and 5 phr of BMIMTFSI showed low values (1.9 and 4.8, respectively) in relation to the NBR/SiO_2_ composite. On the other hand, the NBR compounds with BMIMAlCl_4_ displayed an ΔE parameter of about 20, which confirms changes in the color characteristic of NBR/SiO_2_/BMIMAlCl_4_ systems.

### 3.2. Mechanical Properties, Morphology, Ionic Conductivity, and Thermooxidative Ageing

**Figure 3:**
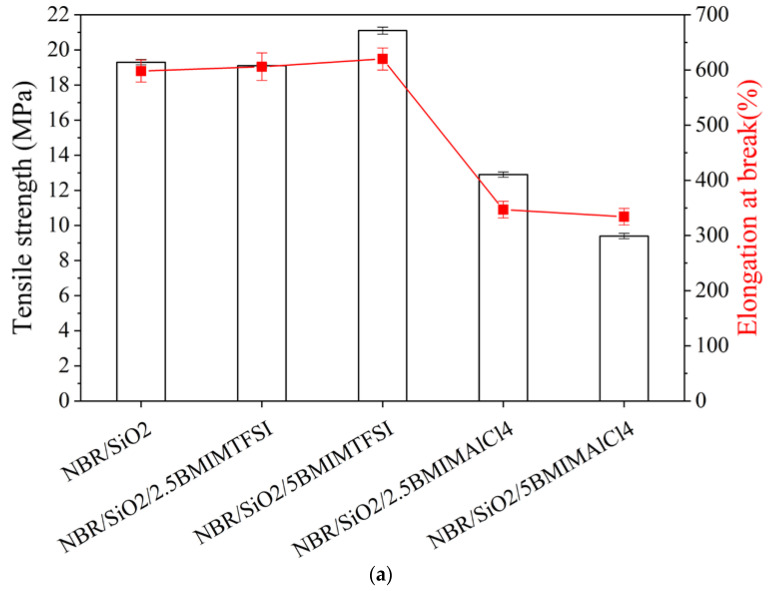

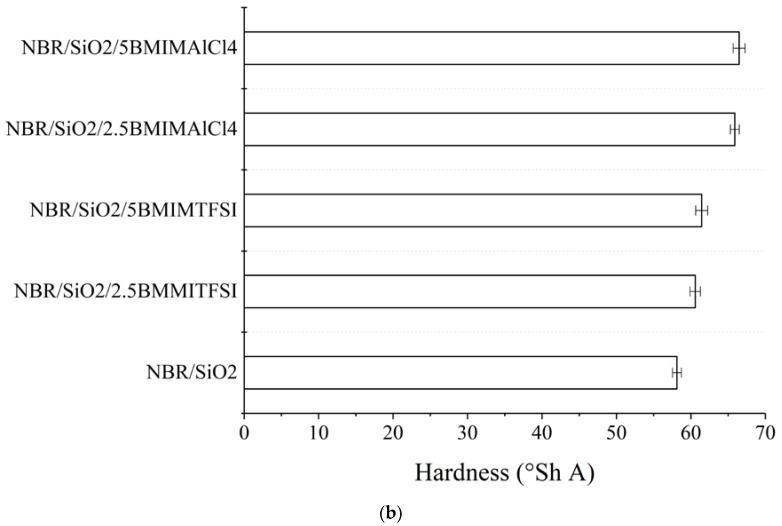
Mechanical properties (**a**) and hardness (**b**) of NBR composites containing BMIMTSFI and BMIMAlCl_4_.

As can be seen in Figure 3a, the presence of the both ILs affected the static-mechanical properties of the silica-filled NBR composites. However, particularly remarkable changes were observed in the case of samples containing IL with AlCl_4_ ions. The addition of 2.5 and 5 phr of hydrophilic BMIMAlCl_4_ in the NBR polymer significantly increased the stiffness of the samples, as shown by the lower elongation at break (Eb). A drop in the tensile strength (TS) value from 19.1 to 9.5 MPa was observed for NBR/5BMIMAlCl_4_ in comparison to the NBR/SiO_2_ reference sample. This was related to the higher values for the crosslink density of the composites and is in agreement with data obtained from swelling experiments and rheometric measurements. Compared to the reference sample, the incorporation of BMIMTFSI ions led to an increase in the Eb parameter, which was related to the higher elasticity and flexibility of the rubber materials. The tensile strength of the NBR sample containing hydrophobic BMIMTFSI was higher than that of the other composites. At a given strain, an increase in stress was noted when 5 phr of BMIMTFSI was added (21.1 MPa), compared to the neat NBR/SiO_2_ (19.1 MPa). The improved mechanical properties of the NBR samples may be related to the reduction of the filler agglomerates and to the enhancement of filler–filler or rubber–filler interactions after the addition of ILs.

As expected, based on crosslinking density measurements, the NBR/SiO_2_/BMIMAlCl_4_ composites also showed the greatest value for hardness, with a figure of 65.5 on the Shore A scale (Figure 3b). Based on the results of tensile tests, the addition of BMIMAlCl_4_ decreased the elasticity and increased the hardness of the vulcanizates in comparison to the reference.

**Figure 4:**
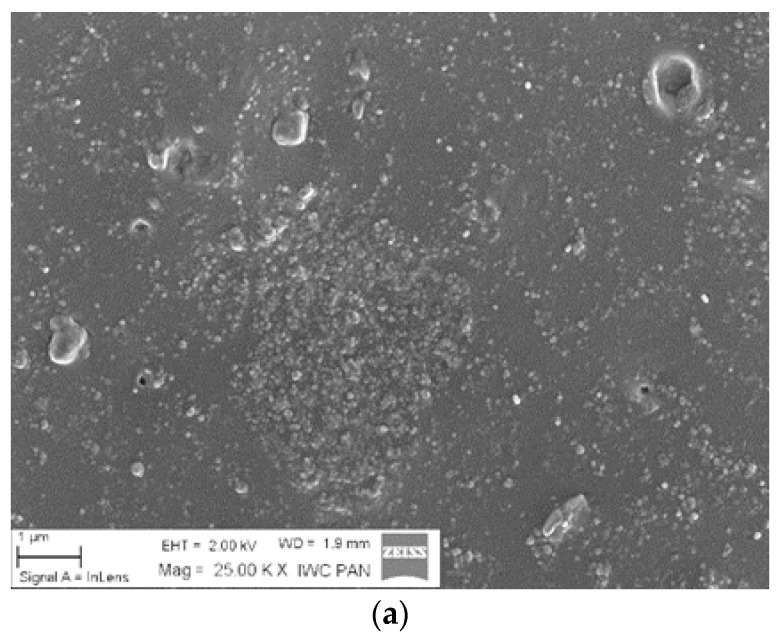

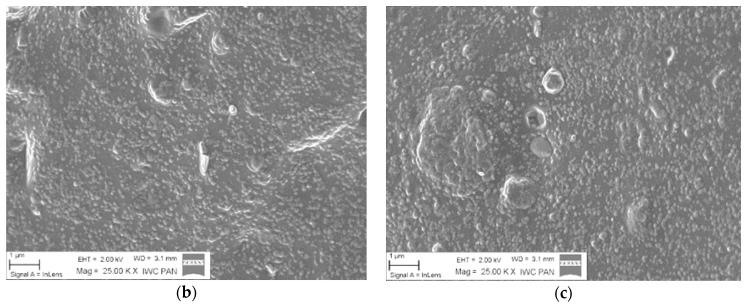
Scanning electron microscopy (SEM) micrographs of NBR/SiO_2_ (**a**), NBR/SiO_2_/5BMIMAlCl_4_ (**b**), and NBR/SiO_2_/5BMIMTFSI (**c**).

The distribution of silica particles in the NBR polymer matrix was analyzed based on SEM micrographs. The presence of numerous silica agglomerates in Figure 4a indicates a poor dispersion of the inorganic hydrophilic filler in the polymer matrix. As shown in Figure 4b,c, the application of 5 phr of ILs significantly decreased the tendency for aggregation and resulted in a more homogenous filler dispersion. In the case of NBR/SiO_2_/BMIMAlCl_4_ samples, the improved filler particle distribution did not lead to mechanical reinforcement in static conditions, because the presence of ILs also catalyzed crosslinking reactions, as shown by the high crosslinking density values.

**Figure 5:**
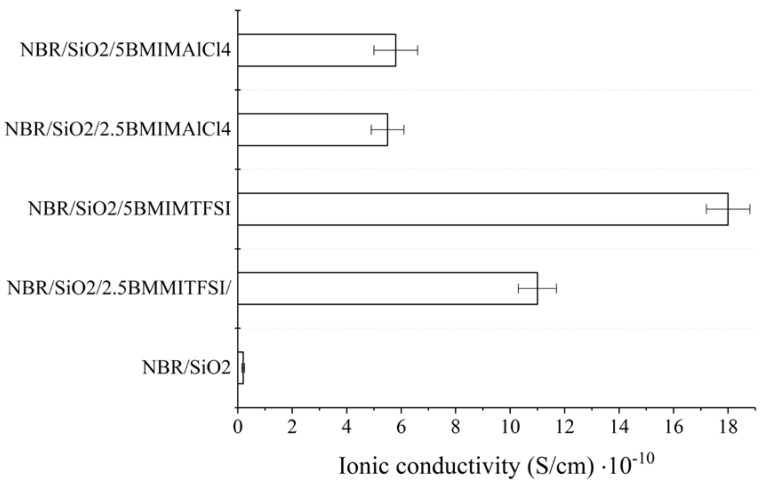
Ionic conductivity of NBR-ionic liquid-based composites measured at room temperature.

The addition of ILs resulted in an increase in the ionic conductivity of the studied composites (Figure 5). The ionic conductivity of the obtained NBR composites is generally dependent on the conductivity and ion concentration of the pure IL. Typical values for IL conductivity measured at 25 °C range from 1.0 to 10.0 mS/cm. Hydrophobic BMIMTFSI has a conductivity of 3.5 mS/cm (25 °C) and the hydrophilic BMIMAlCl_4_ has a conductivity of 9.2 mS/cm (25 °C). The improvement of ionic conductivity in the NBR ionic liquid-based systems is purely based on the contribution of ions [34]. The ionic conductivity values of the NBR/SiO_2_ samples containing 2.5 and 5 phr of BMIMAlCl_4_ increased from 5.6·10^−12^ to 5.5·10^−11^ and 5.8·10^−11^ S·cm^−1^, respectively. Interestingly, the samples that contained BMIMTFSI salt exhibited conductivities of 1.1·10^−9^ S·cm^−1^ (2.5 phr) and 1.8·10^−9^ S·cm^−1^ (5 phr), respectively, at the same concentrations of IL. This observation may be related to the active participation of the AlCl_4_ anions in the crosslinking process or to the lower compatibility of this IL with the hydrophobic polymer.

**Figure 6:**
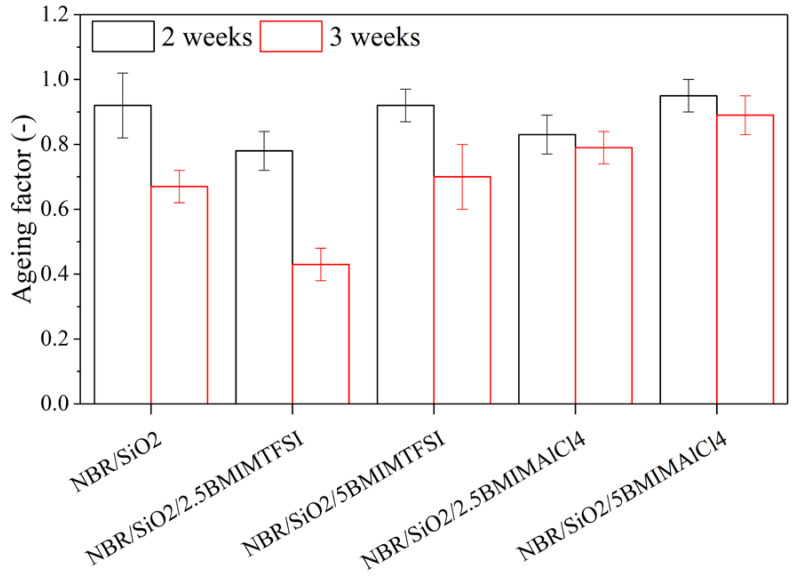
Ageing factor of NBR composites after two and three weeks of thermooxidative ageing.

The static mechanical properties were also measured after thermooxidative ageing over two or three weeks, to calculate the ageing factor K (Figure 6). After two weeks, slight changes in the K parameter value were observed, but the subsequent changes exhibited after three weeks by the samples with BMIMAlCl_4_ were insignificant. It can be concluded that BMIMAlCl_4_ stabilized the rubber mixtures. According to Maciejewska et al. [29], ILs containing chlorides may be assumed to catalyze interfacial reactions, by acting as a catalyst for crosslinking reactions. In the case of BMIMAlCl_4_, the crosslinking density can be attributed to the high efficiency of the reaction between the unsaturated C = C bond in the rubber and the curing agents. The improved ageing stability of the composites containing ILs was most likely related to the limited increase in the crosslink density.

### 3.3. Dynamic-Mechanical Properties (DMA)

**Figure 7:**
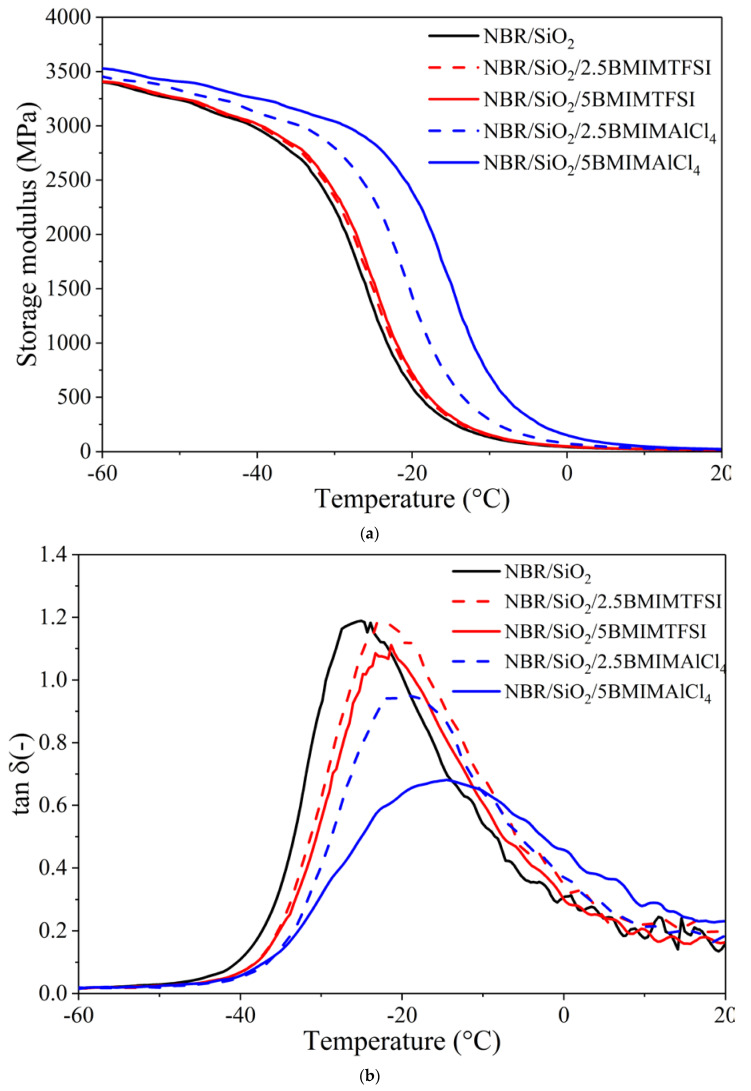
Storage modulus (**a**) and tanδ (**b**) values versus temperature curves for NBR/SiO_2_ composites containing ionic liquids.

Figure 7a,b shows the dynamic-mechanical properties of NBR composites containing ILs, in terms of the storage modulus and loss tangent (tanδ) as functions of temperature. It can be seen that the storage modulus moved toward higher values across the entire temperature range, especially after the application of BMIMAlCl_4_ in the silica-filled NBR system. This shift can be attributed to the strong influence of the ILs on the curing kinetic, as has been previously described by other authors [35,36]. However, it was observed that samples filled with 5 phr of ILs had higher values for the storage modulus, compared to those with 2.5 phr. The reason for this may be the improved dispersion of silica particles in the NBR matrix, due to the higher concentration of ILs, which led to the formation of stronger three-dimensional networks of silica.

In Figure 7b, a single transition can be clearly observed for the NBR-filed vulcanizates, indicating that no microscopic phase separation occurred. The incorporation of hydrophilic BMIMAlCl_4_ caused the tanδ peak height of the NBR vulcanizates to decrease sharply compared to the samples without ILs. The glass transition temperature determined from the position of the tanδ peak is also presented in Table 3, as well as the tanδ peak height. The glass transition temperature determined for the silica-filled NBR without ILs was −25.5 °C, whereas after the application of 2.5 and 5 phr BMIMAlCl_4_, respectively, the T_g_ shifted to −19.1 and −14.5 °C. This may be due to the significantly increased crosslink density of the vulcanizates containing BMIMAlCl_4_, which restricted the mobility of the NBR macromolecules. On the other hand, the application of BMIMTFSI caused slight alterations of T_g_ values and was found, for NBR/SiO_2_/2.5BMIMTFSI and NBR/SiO_2_/5BMIMTFSI, to be −21.4 and −21.2 °C, respectively.

## 4. Conclusions

In this study, silica-filled reinforced elastomer composites were fabricated in the presence of various concentrations of imidazolium salts with different anions. The results from typical rheometric and crosslink density studies showed that the curing efficiency of silica-filled NBR compounds containing BMIMTFSI and BMIMAlCl_4_ was better than that of the reference, as evidenced by the higher torque increment and crosslink density values. The application of 2.5 and 5 phr of ILs did not cause significant alterations in either the mechanical properties or aging stability of the silica-filled NBR composites. On the other hand, a significant increase in ionic conductivity was noted for the NBR/SiO_2_/5BMIMTFSI sample, from 5.6∙10^−^^12^ S/cm for the composite without ILs to 1.8∙10^−9^ S/cm. Most importantly, the selection of the type and load of IL enabled the production of transparent sulfur cross-linked elastomer composites based on a nitrile rubber matrix with silica filler. This work shows that obtaining transparent and reinforced rubber materials based on NBR and silica can be achieved when 2.5 phr of BMIMAlCl_4_ is applied as a dispersing agent. This outcome is impossible to achieve using BMIMTFSI or other concentrations of ILs in NBR/SiO_2_ composite formulations. The results of this study show the great potential of applying ILs to extend the application possibilities of elastomers, especially in transparent sealing systems (seals, hoses, and joints), which can possibly be used for monitoring the flow of oils or hydrocarbons.

## Figures and Tables

**Table 1:** Rheometric parameters of silica-filled NBR composites with different ionic liquids.

Composite Name	M_min_ (dNm)	ΔM (dNm)	τ_02_ (min)	τ_90_ (min)
NBR	2.17	20.55	1.31	32.96
NBR/SiO_2_/2.5BMIMTFSI	2.04	19.42	1.87	33.14
NBR/SiO_2_/5BMIMTFSI	1.87	18.38	1.79	34.98
NBR/SiO_2_/2.5BMIMAlCl_4_	1.94	27.09	0.57	6.97
NBR/SiO_2_/5BMIMAlCl_4_	3.25	34.15	0.42	3.49

M_min_—minimum torque moment; ΔM—torque increment; τ_02_—scorch time; τ_90_—optimal curing time.

**Table 2:** Changes in the color parameters of the NBR composite after the addition of ionic liquids at different concentrations.

Composite Name	L	a*	b*	ΔE
NBR/SiO_2_	69.5	6.9	24.4	-
NBR/SiO_2_/2.5BMIMTFSI	69.6	6.8	26.1	1.9
NBR/SiO_2_/5BMIMTFSI	71.5	4.8	19.4	4.8
NBR/SiO_2_/2.5BMIMAlCl_4_	53.7	13.9	35.6	20.4
NBR/SiO_2_/5BMIMAlCl_4_	65.6	16.5	55.5	32.8

L—lightness; a*—red-green coordinate; b*—blue-yellow coordinate; ΔE—total color change.

**Table 3:** Glass transition temperatures and the tanδ peak for NBR/SiO_2_ composites containing ionic liquids.

Composite Name	T_g_ (°C)	tanδ_max_ (–)
NBR/SiO_2_	−25.5	1.2
NBR/SiO_2_/2.5BMIMTFSI	−21.4	1.2
NBR/SiO_2_/5BMIMTFSI	−21.1	1.1
NBR/SiO_2_/2.5BMIMAlCl_4_	−19.1	0.9
NBR/SiO_2_/5BMIMAlCl_4_	−14.5	0.6

T_g_—glass transition temperature; tanδ_max_—maximum of the tanδ value.
